# Target trial emulation in oncology: current use and future directions

**DOI:** 10.1186/s40779-026-00685-9

**Published:** 2026-02-24

**Authors:** Hui-Yao Huang, Le Wang, Sheng Xu, Shuo-Peng Jia, Dan-Dan Kong, Xue-Jing Zhang, Si-Qi Wang, Han-Qing He, Hao-Ran Chen, Lu-Zhu Xia, Lan-Wei Guo, Yu Tang, Ling-Bin Du, Ning Li

**Affiliations:** 1 Clinical Trials Center, National Cancer Center/National Clinical Research Center for Cancer/Cancer Hospital, Chinese Academy of Medical Sciences and Peking Union Medical College, Beijing, 100021 China; 2 Department of Cancer Prevention, Zhejiang Cancer Hospital, Hangzhou Institute of Medicine (HIM), Chinese Academy of Sciences, Hangzhou, 310022 China; 3 Global Statistics and Data Science, BeiGene, Shanghai, 200001 China; 4 School of Statistics, Renmin University of China, Beijing, 100872 China; 5 School of Public Health, Qingdao University, Qingdao, 266071 Shandong China; 6 Minzu University of China, Beijing, 100081 China; 7 Department of Clinical Research Management, the Affiliated Cancer Hospital of Zhengzhou University & Henan Cancer Hospital, Zhengzhou, 450008 China

**Keywords:** Target trial emulation (TTE), Oncology, Bias, Real-world database

## Abstract

Target trial emulation (TTE) has demonstrated popularity because of its ability to improve the reliability of causal inference from observational data. Nevertheless, knowledge about the current use, potential challenges, and insights of target trials in oncology is scarce. A total of 90 TTE studies in cancer areas were identified through systematic reviews in PubMed and Embase. Among the 54 applications in cancer treatment, registry databases (44.4%) and overall survival (OS, 63.0%) were predominantly used as data sources and primary endpoints, respectively. Approximately 30 (55.6%) of the included TTE cases were associated with immortal time bias, and 21 (38.9%) were associated with prevalent user bias. Among the 21 trials from 13 studies that aimed to calibrate the results from preexisting randomized controlled trials (RCTs), only 42.9% met both statistical agreement and estimate agreement. The availability of fit-for-purpose data sources and uncertainty about result concordance were the main hurdles limiting the quantity and quality of TTE in oncology areas. Promoting regulatory acceptance by initiating special projects could be crucial for the expanded application of real-world data (RWD) using TTE. Potential solutions, such as the integration of electronic medical records at the regional or country level, linkage with insurance claims databases, the modernization of eligibility criteria, the use of OS as the primary endpoint, and other best practices, were recommended for improving the feasibility and quality of oncology TTE.

**Supplementary Information:**

The online version contains supplementary material available at 10.1186/s40779-026-00685-9.

## Background

Randomized controlled trials (RCTs) are the gold standard for estimating the causal effects of medication on health outcomes. However, their high cost, limited generalizability, and constrained scope mean that RCTs cannot address all clinically relevant questions, particularly in oncology [1]. There is a global need for robust evidence generation of marketed cancer medicines using observation studies when big data becomes available, informing best clinical practice and policy decisions [2]. Research questions about real-world or long-term risk-benefit, optimal population, treatment sequences, comparative effectiveness, and cost-effectiveness of approved regimens are typically addressed through analyses of observational data [3]. This is especially true in oncology, where innovative drugs are proliferating along with the most frequent use of expedited programs in regulatory approval, which leaves more gaps to fill [4].

Cancer is highly heterogeneous and characterized by diverse biological behaviors, multiple comorbidities, and complex treatment pathways. Patients frequently experience complications such as infection, thrombosis, cachexia, treatment-related toxicity, and psychological distress, which further complicate the clinical course and management [5, 6]. RCTs often enroll a highly selected subset of patients with favorable performance status and limited comorbidities, which limits their external validity [7]. Conducting RCTs for every relevant clinical question in oncology is therefore neither feasible nor ethical, especially for rare cancers, elderly or frail populations, or for rapid post-approval evidence generation [8]. Observational studies using insurance claims, electronic health records (EHRs), and registries play a critical complementary role. However, causal inference from observational data is challenging because of several potential biases, such as selection bias, confounding bias, immortal time bias, and prevalent user bias [9].

Controlling bias in observational analyses by designing them according to the principles of RCTs has long been proposed to answer causal questions of interest. Until 2016, when Hernán and Robins [10] proposed a structured and coherent framework known as target trial emulation (TTE), investigators were increasingly designing observational studies based on hypothetical RCTs, including in oncology areas [11]. This is because the target trial framework could not only potentially avoid common biases in observational studies but also enhance the transparency of design, analytics, and reporting, as well as the interpretation of effect estimates similar to those of RCTs. Nevertheless, knowledge is scarce regarding TTE use, challenges, and solutions in oncology. This study introduces key TTE components with a case study; presents systematic review findings; discusses challenges, solutions, and recommendations for oncology applications; and specifically aims to evaluate the current use of TTE in oncology, identify practical challenges, and provide guidance to optimize its application in observational cancer research.

## Key components of TTE

TTE involves 2 main steps. The first step involves clearly defining a causal question via the specification of the target trial protocol, including eligibility criteria, treatment strategies, assignment procedures, follow-up period, outcomes, causal contrasts, and analysis plan. The second step involves emulating the target trial as closely as possible by using fit-for-purpose datasets [11]. The fit-for-purpose dataset refers to a dataset with relevance and reliability. Relevance includes the availability of key data elements (exposures, outcomes, covariates) and a sufficient number of representative patients for the study, whereas reliability is focused on data accuracy, completeness, provenance, and traceability [12]. In oncology, this typically involves integrating structured clinical information (stage, biomarkers, treatment regimens) from EHRs with registry or claims data to ensure both data quality and longitudinal continuity. For example, the epidemiological strategy and medical economics metastatic breast cancer (ESME-MBC) study emulated a trial (NCT00028990) comparing paclitaxel versus paclitaxel plus bevacizumab in human epidermal growth factor receptor 2 (HER2)-negative metastatic breast cancer [13, 14]. The details are presented in Additional file 1: Table S1. A registry database from a retrospective cohort was identified as the fit-for-purpose data source.

A critical aspect of TTE is aligning eligibility, treatment assignment, and follow-up at time zero [10, 11]. Each participant’s time zero must correspond to the moment when he or she meets the eligibility criteria and is assigned to a treatment strategy. This is easy to control in clinical trials but complicated in observational studies [10, 11]. Studies lacking synchronization of eligibility, treatment assignment, and follow-up can lead to 4 types of failures, resulting in related biases (Additional file 1: Fig. S1). Taking the ESME-MBC study as an example, eligibility was defined as the time of metastatic breast cancer diagnosis, and the start of follow-up was the initiation of treatment. Patients initiated paclitaxel or bevacizumab as first-line treatment within a short period, defined as 1 month before to 4 months after the metastatic diagnosis. That is, patients had to survive for 3 months from the start of follow-up and to receive the assigned treatment, and post-treatment information was used for patients who initiated treatment before the diagnosis. Selection bias may arise because of potential imbalances in confounding between treatment and control groups. This may occur because of the non-randomized nature of observational studies, especially for studies including individuals based on post-treatment criteria (e.g., individuals who did not experience an outcome before the start of follow-up). Moreover, immortal time bias may arise because patients could potentially receive treatment during an uncertain period after diagnosis, which requires them to survive during this time [15].

## Current use of TTE in oncology

Through a systematic literature review (PROSPERO: CRD42025587232) of PubMed and Embase from inception to September 30, 2025 (Additional file 1: Tables S2 and S3; Additional file 1: Fig. S2), we identified a total of 90 oncology TTE applications, including 54 studies on treatment and 36 studies on prevention or supportive care. Notably, the number of related research has increased dramatically (Additional file 1: Fig. S3). A detailed analysis of the 54 applications in cancer treatment was further conducted (Table 1).

**Table 1:** Characteristics of included target trial emulation on cancer treatment

Characteristics	Number (%)
Location of participants	
North America	23 (42.6)
Europe	16 (29.6)
Asia	10 (18.5)
Oceania	1 (1.9)
International	4 (7.4)
Data source	
Registry	24 (44.4)
Multiple linked databases	13 (24.1)
Electronic health record	10 (18.5)
Health insurance	7 (13.0)
Protocol registration	
Yes	4 (7.4)
No	50 (92.6)
Key components listed in the table	
Yes	35 (64.8)
No	19 (35.2)
Emulation context	
No available RCTs	32 (59.3)
Calibrated against RCTs evidence	22 (40.7)
Treatment evaluated	
Drug	31 (57.4)
Surgery	13 (24.1)
Combination	6 (11.1)
Radiology	3 (5.6)
Radioembolization	1 (1.9)
Outcome	
OS	34 (63.0)
Multiple outcomes	16 (29.6)
Local recurrence-free survival	1 (1.9)
Time to next treatment	1 (1.9)
Safety	1 (1.9)
PFS	1 (1.9)
Inconsistent time zero	
Follow-up starts at eligibility, but treatment is assigned later	25 (46.3)
Follow-up starts after eligibility criteria completion and treatment assignment	14 (25.9)
Follow-up starts before treatment assignment and eligibility	7 (13.0)
Follow-up starts at eligibility, but after treatment assignment	5 (9.3)
Not reported	3 (5.6)
Potential bias	
Immortal time bias and misclassification of treatment	25 (46.3)
Prevalent user bias	14 (25.9)
Prevalent user bias and selection bias due to posttreatment eligibility	7 (13.0)
Immortal time bias and selection bias due to posttreatment eligibility	5 (9.3)
Not reported	3 (5.6)
Confounding adjustment	
Inverse probability weighting	26 (48.1)
Propensity score	8 (14.8)
Multivariate regression	4 (7.4)
Combination	16 (29.6)
Causal contrast of interest	
Per protocol	34 (63.0)
Intention-to-treat	14 (25.9)
Both	6 (11.1)

*RCTs* randomized controlled trials, *OS* overall survival

Generally, study hypotheses determine the innovativeness, and the data source used determines the confidence of the study results. With respect to all the TTE studies of cancer treatment, 32 (59.3%) studies aimed to generate novel evidence in the absence of RCTs, and only 22 (40.7%) articles aimed to calibrate or extend the results from pre-existed RCTs. Moreover, registry databases (44.4%) were predominantly used as observational data sources, whereas multiple linked databases were used in 13 (24.1%) studies, followed by EHRs and health insurance claims. From the perspective of transparency, only 4 studies preregistered the intended research protocol [16–19]. Overall survival (OS) was predominantly used as the primary outcome. More information on bias and confounding adjustment is presented in Table 1.

## Agreement between TTE and RCTs

**Fig. 1:**
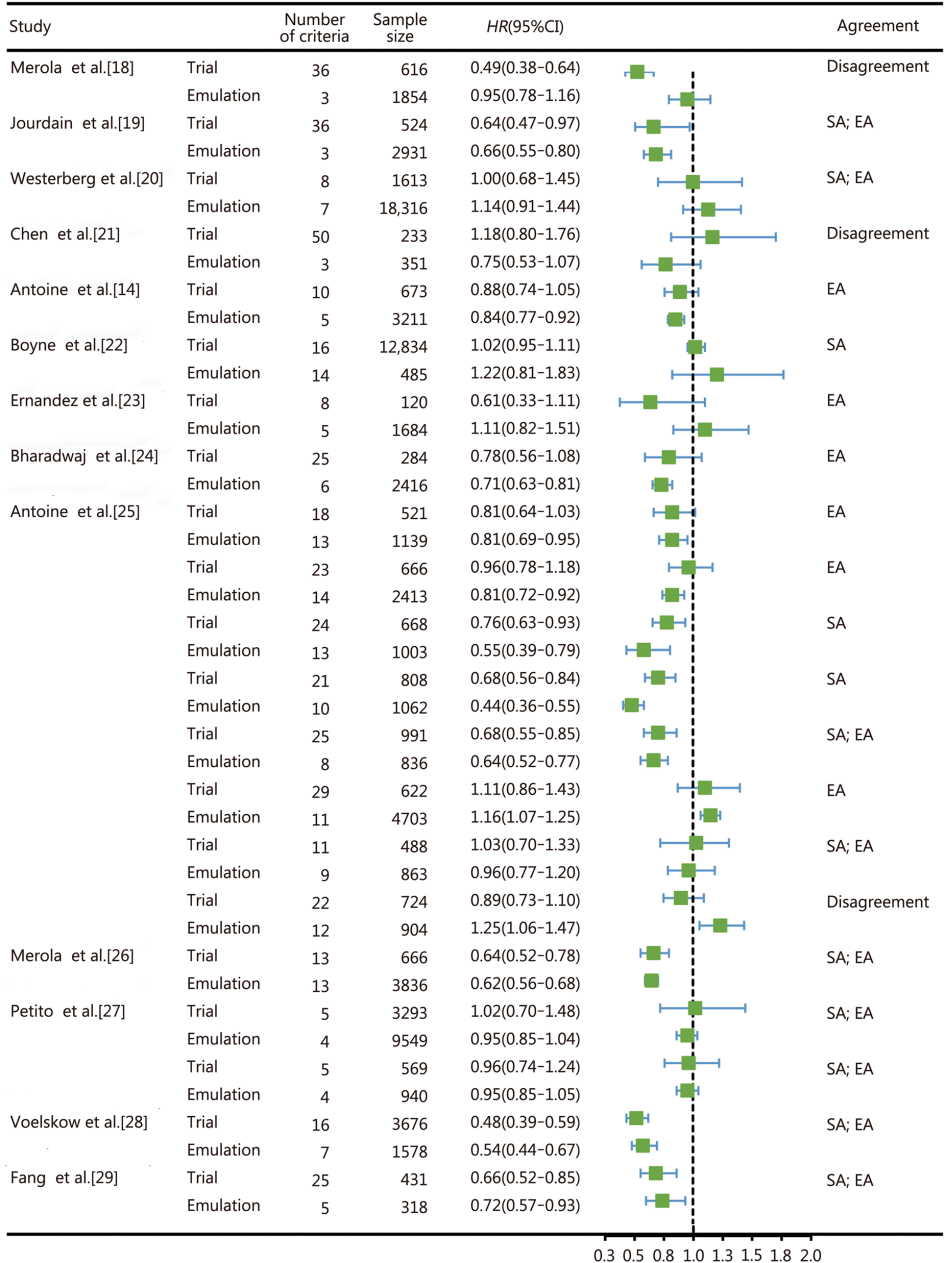
Effect estimates and agreement metrics of the 21 calibrated oncology trials. The green box denotes the point estimate of the HR, and the horizontal line represents the 95% CI. Estimate agreement was defined as estimates for the trial emulation falling within the 95% CI for the trial results, and statistical agreement was defined as the estimates and CIs on the same side of the null for both emulation and trial. EA estimate agreement, SA statistical agreement, HR hazard ratio, CI confidence interval

Among 22 articles calibrating against RCTs (Additional file 1: Table S4), the overall emulation rate of inclusion and exclusion criteria was 40.1%, with 228 of the 568 conditions successfully emulated. Further evaluation on the estimate agreement [estimates for the trial emulation fell within the 95.0% confidence interval (CI) for the trial results] and statistical agreement (estimates and CIs on the same side of the null) were performed for 21 TTEs out of 13 articles with available data [14, 18–29], according to the RCT-DUPLICATE framework [30]. Only 9 TTEs (42.9%) met both statistical agreement and estimate agreement criteria, 6 (28.6%) only met estimate agreement criteria, 3 (14.3%) trials only met statistical agreement criteria, and 3 (16.7%) met neither (Fig. 1) Studies that met dual-agreement criteria exhibited higher emulation rates than those not (median 80.0% vs. 52.0%), suggesting that closer alignment with RCTs eligibility criteria may increase the likelihood of achieving concordant results.

Although the reproducibility of oncological TTE studies remains under evaluation, it is important to recognize that no explicit regulatory thresholds for TTE acceptance have been established. The observed dual-agreement rate of 42.9% among the above 21 TTEs represents low reproducibility under stringent criteria. For comparison, the RCT-duplicate initiative reported that 21 out of 32 (65.6%) trial emulations across multiple disease areas achieved dual agreement [30]. Several factors may explain the lack of agreement observed in the remaining studies. Incomplete emulation of eligibility criteria, unstandardized treatment sequencing, and heterogeneous data quality across sources likely contributed to discordant findings. In addition, variations in endpoint definitions and follow-up durations between emulated and original trials may further limit comparability. To improve concordance, future TTE research should enhance emulation fidelity through transparent reporting, strengthen adjustment for unmeasured confounding using advanced causal inference methods, and promote harmonized definitions of treatment and outcome measures across databases to better align real-world emulations with trial protocols.

## Potential challenges and solutions for oncology TTE

**Fig. 2:**
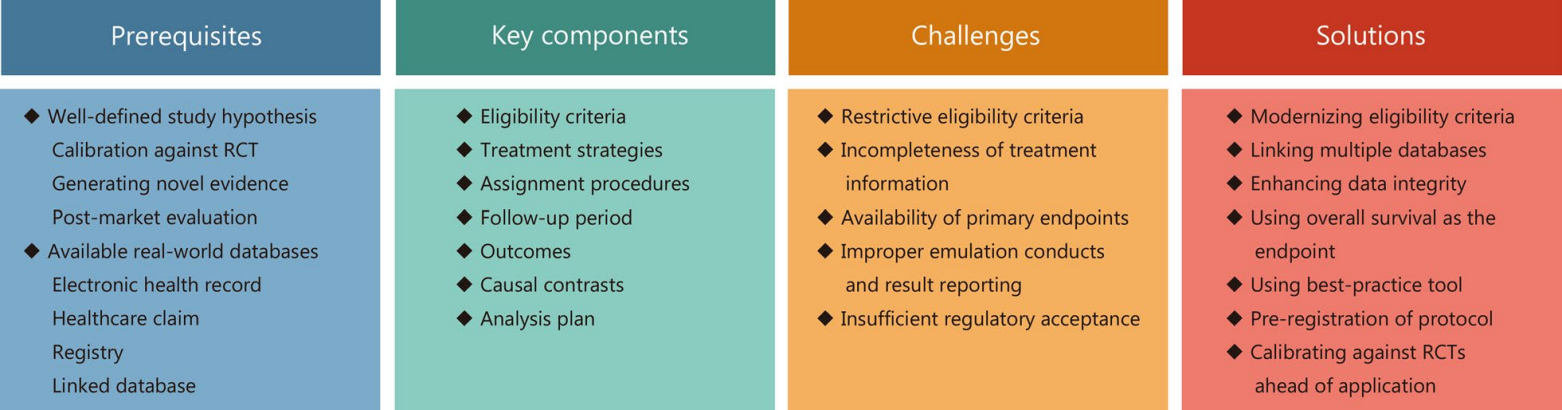
Potential challenges and solutions for TTE in oncology based on key components. RCTs randomized controlled trials, TTE target trial emulation

Given the methodological advantage of TTE and the urgent need for high-quality real-world evidence to inform best clinical practice in oncology, our findings of limited evidence and uneven distribution between regions inspired us to further consider the underlying reasons. We believe that the accessibility of fit-for-purpose data sources is the most critical factor. Specifying all the elements of TTE cannot eliminate the inherent bias of observational studies and facilitate reliable causal inference alone. The success of this approach requires access to fit-for-purpose data sources that contain all the information with sufficient quality needed for emulating related oncology target trials. This includes the availability of data for key study components (eligibility criteria, exposures, outcomes, covariates), sufficient representative patients for the study, and the accuracy, completeness, and traceability of the source data. Compared with other disease areas, the fit-for-purpose data required for trial emulation are particularly difficult for oncology because of the following challenges (Fig. 2).

### Restrictive eligibility criteria

Oncology studies often apply restrictive eligibility criteria, including not only sociodemographic characteristics and medication information but also numerous clinical variables, such as diagnosis and disease history, comorbidities, organ function, and viral infection [31, 32]. This makes it almost impossible for insurance claims databases and highly challenging for EHR to capture most of the related information at baseline to independently identify a population comparable to the emulated trial. For example, 1 identified study could only emulate 8.0% of trial eligibility conditions because current health status and comorbidities were unavailable in its EHR database, and another failed to operationalize laboratory screening requirements [18, 20]. Consequently, TTE studies based on registries and linked databases achieved a median emulation rate of 62.0%, compared to 20.0% for those relying exclusively on health care claims and EHRs (Additional file 1**: Table S4**). This is why resource-consuming registry databases collected for scientific purposes remain the most common data source for identified oncology TTE, and the emulation rate of the involved eligibility criteria was less than half in the case of highly selected trials. Modernizing eligibility criteria appropriately and transparently could represent an effective solution, redefining non-routinely measured variables using validated surrogate markers (e.g., laboratory thresholds and diagnostic codes), prioritizing core variables essential for treatment assignment, and ensuring transparent justification for any adaptation [31, 32]. Although modernizing criteria can expand real-world inclusiveness and enhance external validity by reflecting broader patient populations, excessive simplification may risk diluting causal interpretability. Therefore, a balanced approach that aligns data availability with clinical rationale is essential to ensure both feasibility and scientific validity in future TTE applications.

### Incompleteness of treatment strategies

In oncology, treatment exposure is often dynamic and continuous [33]. Well-documented treatment strategies are generally accessible through insurance databases, whereas information on sociodemographic and clinical confounders is typically obtained from medical records. Integrating data from multiple linked sources, such as registries and insurance claims, can substantially improve treatment completeness and patient follow-up consistency in identified successful emulations. Key variables generally include patient identifiers, dates, treatment codes, and diagnostic information, which together allow for accurate reconstruction of treatment trajectories and outcomes. To address privacy concerns, privacy-preserving record linkage techniques can facilitate cross-database integration while maintaining data confidentiality.

### Availability of primary endpoints

Many oncology TTE studies could not emulate trials with imaging-based endpoints because of fragmented hospital data [18, 20]. Inconsistency of imaging assessment and reporting in clinical practice could even worsen the situation and preclude accurate assessment. Using OS as an endpoint could be more feasible and reliable when linked to the mortality database [33]. This is why the OS was the primary endpoint in more than 60.0% of the identified emulations. Expanding the integration of EHRs systems at the regional or national level may improve endpoint completeness and patient travel traceability.

### Potential biases and analytical approaches

Although TTE provides a structured framework to estimate causal effects using observational data, it is not without methodological limitations. A critical challenge is the precise definition of time zero, which represents the start of follow-up or treatment initiation. In real-world datasets, variability in treatment initiation, delays in recording, or prior exposures can create ambiguity, leading to immortal time bias or misalignment between exposure and outcome assessment. In our systematic review, 30 (55.6%) of the included TTE studies suffered from immortal time bias, highlighting the practical impact of a misdefined time zero on causal inference. To address this, a standardized and reproducible approach can be applied. Hierarchical rules may be established to prioritize clinically relevant events (e.g., diagnosis date, first treatment, and registry enrollment) and resolve conflicts. Automated algorithms can extract candidate dates from structured data (diagnoses, treatments) and unstructured sources (clinical notes) using natural language processing. Linking multiple data sources ensures completeness and allows cross-validation. Sensitivity analyses using alternative definitions of time zero can assess robustness, whereas preregistration of time zero definitions and analytic workflows enhances transparency and reproducibility.

TTE also relies on causal inference methods, such as inverse probability weighting or clone-censor-weight approaches, to adjust for confounding. These methods require key assumptions, including no unmeasured confounding, correct model specification, and positivity, any violation of which may bias the effect estimates. This limitation is reflected in our review, where only 42.9% of the studies achieved dual agreement with the corresponding RCTs, indicating that methodological constraints can influence agreement outcomes. Other potential biases include selection bias, measurement error, and incomplete capture of eligibility criteria or treatment strategies. Across the studies we reviewed, the overall emulation rate of the eligibility criteria was 40.1%, demonstrating that more than half of the trial-defined conditions could not be fully replicated using observational data. To mitigate these issues, we recommend careful study design, rigorous assessment of data quality, pre-specification of analysis plans, transparent reporting, and sensitivity analyses to examine robustness to key sources of bias. These strategies can enhance the validity and interpretability of TTE results in oncology.

### Improper emulation conduct and reporting

Despite these challenges, investigators have shown a significant increase in enthusiasm for TTE. Some quality issues related to oncology trial emulation should be considered. First, for the 32 identified emulations with no available RCTs, benchmarking against existing trial results was not conducted beforehand. To increase confidence in the results, calibrating against existing trials before extending the observational analysis to answer a different question is recommended whenever available [34]. Second, 30 (55.5%) of the included TTE cases were associated with immortal time bias, and 21 (38.9%) were associated with prevalent user bias. This finding reminds us that TTE is not a magic bullet and that the corresponding results may still be fragile because of the lack of randomization and blinding and the typical use of retrospective data. Best practices, such as using benchmarking against existing RCTs when possible, applying clone-censor-weight methods for time-varying exposures, using open tools such as CERBOT for protocol standardization, and conducting robustness assessment using deterministic and probabilistic sensitivity analyses, should be prioritized to ensure methodological rigor and transparency [35].

Transparency is crucial for applying TTE to oncology. Owing to the relative complexity of oncology research, changes in key components of TTE may lead to terminations or modifications of the study [36, 37]. Multiple revisions to the study design and multiple tests before the disclosure of the results may lead to false-positive results. However, the study revealed that only 4 TTE studies preregistered the protocol. To maintain transparency, the preregistration of the protocol and statistical plan, as well as the disclosure of modifications, are recommended.

### Insufficient regulatory acceptance

Despite growing methodological interest, the expanded application of TTE in oncology to support regulatory decision-making remains limited because of insufficient regulatory acceptance [38, 39]. A key challenge is the absence of defined regulatory standards or thresholds for acceptable replication success. Our analysis revealed that only 42.9% of oncology TTE achieved dual agreement with RCT results, a level of reproducibility that is lower than the approximately 65.0% dual-agreement rate reported by the RCT-DUPLICATE initiative across non-oncology indications [30]. Although no formal benchmark has been established, these findings suggest that current oncology TTE do not yet meet the level of evidentiary consistency likely required for regulatory confidence.

To enhance robustness and facilitate regulatory acceptance, researchers can take several proactive steps beyond waiting for initiatives such as ENCORE, which was initiated by the U.S. Food and Drug Administration in September 2024 [40]. First, prespecifying and registering detailed emulation protocols can increase transparency and reduce selective reporting, although requirements by academic journals (such as the TARGET checklist) and regulatory expectations for protocol registration when TTE evidence informs decision-making [36]. Second, benchmarking TTE findings against existing RCTs when available provides empirical calibration of bias. Third, replicating analyses across independent data sources and conducting probabilistic sensitivity analyses can help quantify uncertainty and strengthen confidence in causal inference. These efforts, coupled with ongoing regulatory calibration projects such as ENCORE, may accelerate the establishment of oncology-specific standards for real-world evidence evaluation and pave the way for broader regulatory adoption of TTE methodologies.

## Role positioning of TTE in oncology

### When to use TTE

The TTE framework offers a promising paradigm to strengthen causal inference from observational data by explicitly aligning study design and analysis with the principles of RCTs. In oncology, TTE plays an increasingly important role in generating timely, cost-efficient, and more representative evidence, particularly when RCTs are not feasible because of ethical, logistical, or financial constraints. It enables the exploration of underrepresented subgroups, post-marketing comparative effectiveness, long-term safety, and optimal sequencing of therapeutic regimens, questions that traditional RCTs seldom address.

### When not to use TTE

Nevertheless, TTE has inherent limitations in oncology. Specific limitations arise when treatment strategies are ill-defined, involve multiple versions, or are not practically implementable, as seen in adaptive chemotherapy regimens or sequential multiline therapies, where the consistency assumption may be violated. Similarly, efforts to emulate extreme or unrealistic strategies, such as treating all versus treating none or enforcing universal early initiation, can breach the positivity or overlap assumption, resulting in unstable effect estimates. Interference or spillover effects, for example, those introduced by hospital-level changes in oncological care pathways, may further violate the stable unit treatment value assumption, thereby complicating causal interpretation. In addition, mediation analyses remain constrained by cross-world assumptions, even when conducted within a TTE framework. Additional file 1: Table S5 summarizes these scenarios, the associated methodological challenges, and potential alternative analytical approaches relevant to oncology research.

## Limitations and future directions

This study has several limitations. First, the design quality and data sources of the included TTE were heterogeneous, which may have affected the estimated emulation rates and agreement with the RCTs. Second, we analyzed only published studies indexed in English databases, which may have led to publication bias. Third, the absence of standardized reporting for TTE in oncology may have limited the comprehensiveness of our assessment. Future research should focus on establishing standardized reporting guidelines for oncology TTE, integrating multisource RWD to improve completeness, and developing benchmarking frameworks to validate emulated results against RCTs. Strengthening methodological transparency and cross-regional collaboration will be critical to enhancing the regulatory and clinical value of TTE in oncology.

## Conclusions

The application of TTE in oncology has significantly increased, but the overall quantity and quality are still limited. This could be largely constrained by the availability of fit-for-purpose data sources, considering the unique challenge of oncology trial emulation, as well as by the uncertainty of concordance in results. Promoting regulatory acceptance by initiating projects to calibrate real-world evidence studies in oncology against RCTs could be crucial for all the expanded applications of real-world databases using TTE. The integration of electronic medical records at the regional or national level and linkages with insurance claim databases are essential for improving the feasibility and quality of oncology TTE. Modernizing eligibility criteria appropriately and using OS as the primary endpoint could also be helpful. The study also emphasized that although the target trial offered a unified framework to leverage non-interventional data to answer questions of interest in a causal inference manner, TTE is not a magic bullet, and that corresponding results still suffer from biases. Best practices for high-quality trial emulations in oncology should be adopted.

## Supplementary Information

**Additional file 1**. Full list of included studies. **Table S1** Key components and challenges for target trial emulation in oncology. **Table S2** Search strategy in PubMed. **Table S3** Search strategy in Embase. **Table S4** Characteristics of 22 studies aimed to calibrate or extend the results from pre-existed RCTs. **Table S5** When not to use target trial emulation. **Fig. S1** Biases caused due to asynchronization of eligibility, treatment assignment, and follow-up. **Fig. S2** PRISMA flowchart. **Fig. S3** Time trends in publications using target trial emulation in oncology.

## Data Availability

The datasets used and analyzed during the current study are available from the corresponding author upon reasonable request.

## Abbreviations

EHRs: Electronic health records
OS: Overall survival
RCTs: Randomized controlled trials
RWD: Real-world data
TTE: Target trial emulation
