# Supplementary material for: Target trial emulation in oncology: current use and future directions
Source: Mil Med Res. 2026 Feb 24;12:99. doi: 10.1186/s40779-026-00685-9 (PMC12930863; doi:10.1186/s40779-026-00685-9)
Supplement: Supplementary file 1 — Additional file 1. Full list of included studies. Table S1 Key components and challenges for target trial emulation in oncology. Table S2 Search strategy in PubMed. Table S3 Search strategy in Embase. Table S4 Characteristics of 22 studies aimed to calibrate or extend the results from pre-existed RCTs. Table S5 When not to use target trial emulation. Fig. S1 Biases caused due to asynchronization of eligibility, treatment assignment, and follow-up. Fig. S2 PRISMA flowchart. Fig. S3 Time trends in publications using target trial emulation in oncology. [file 40779_2026_685_MOESM1_ESM.pdf]

Full list of included studies

A total of 90 studies were included in our review, including 54 studies regarding cancer treatment [1-54], 33 studies regarding cancer prevention and screening [55-87], and 3 studies regarding supportive care or surveillance [88-90].

Table S1 Key components and challenges for target trial emulation in oncology

Components	Description	Target trial	Emulation trial	Key challenges
Eligibility criteria	Who will be included in the study?	Histologically or cytologically confirmed adenocarcinoma of the breast, locally recurrent disease, with 43 criteria	Same as trial, but 33 items without information	Certain eligibility criteria might not be available or identified with sufficient accuracy in RWD
Treatment strategies	What interventions will eligible persons receive?	Paclitaxel with or without bevacizumab as first-line treatment	Same as trial	Information on treatment administration or the use of concomitant or supportive therapies might not be complete or accurate
Assignment procedures	How will eligible persons be assigned to the interventions?	Randomization	Based on observed treatment strategies	Observed treatment strategies might be influenced by measured or unmeasured confounders
Follow-up period	During which period will eligible persons be followed in the study?	Begins at randomization	Starts at treatment initiation	Designation of the index date should be consistent, and the duration of follow-up periods should be comparable across arms
Outcomes	What outcomes in eligible persons will be compared among intervention groups?	Primary endpoints: progress-free survival;<br>Secondary endpoints: OS, objective response rate, toxic effects, quality of life	OS	Whether endpoints used can be reliably and consistently measured in route practice
Causal contrasts	Which counterfactual contrasts will be estimated using the above data?	Intention-to-treat for efficacy;<br>Per protocol for toxic effects	Real-world analogue of the intention-to-treat	Potential bias may be introduced by the analogue, due to various confounders and the difference in follow-up start time between the hypothetical and emulation trial
Analysis plan	How will the counterfactual contrasts be estimated?	Kaplan-Meier analysis and Cox proportional hazard model	Same. Confounding covariates were adjusted, and sensitivity analyses were done	Intensive sensitivity analyses are required for the assessment of model assumptions, the unmeasured confounders, misclassification and missingness

RWD real-world data, *OS* overall survival

Table S2 Search strategy in PubMed

Number	Strategy
1	trial emulat*
2	“target trial”
3	emulat* trial
4	pseudotrial*
5	emulat* analysis
6	“comparative effectiveness”
7	“causal analysis*”
8	“causal inference”
9	“observational study” or “observational data”
10	real-world or “real world” or RWD
11	cohort or case-control or “case control” or cross-sectional or “cross sectional”
12	“electronic medical record” or “electronic health record”
13	“routinely-collected data”
14	database
15	register*
16	“medical claim” or insurance
17	Neoplasms[MeSH Terms]
18	cancer or neoplasm* or carcinoma or oncology
19	1 or 2 or 3 or 4 or 5 or 6 or 7 or 8
20	9 or 10 or 11 or 12 or 13 or 14 or 15 or 16
21	17 or 18
22	“2010/01/01”[Date - Publication]: “2025/09/30”[Date - Publication]
23	19 and 20 and 21 and 22

RWD real-world data

Table S3 Search strategy in Embase

Number	Strategy
1	trial emulat*
2	‘target trial’
3	emulat* trial
4	pseudotrial*
5	emulat* analysis
6	‘comparative effectiveness’:ab,ti
7	‘causal analysis*’
8	‘causal inference’
9	‘observational study’ or ‘observational data’
10	real-world or ‘real world’ or rwd
11	cohort OR ‘case control’ OR ‘cross sectional’
12	‘electronic medical record’ or ‘electronic health record’
13	‘routinely-collected data’
14	database
15	register*
16	‘medical claim’ or insurance
17	malignant neoplasm’/exp
18	cancer or neoplasm* or carcinoma or oncology
19	1 or 2 or 3 or 4 or 5 or 6 or 7 or 8
20	9 or 10 or 11 or 12 or 13 or 14 or 15 or 16
21	17 or 18
22	[2010-2025]/py
23	#17 AND #18 AND #21 AND [embase]/lim AND [2010-2025]/py
24	#17 AND #18-AND #21 AND smbaseVlimAND [01-01-20251/sdNOT [01-10-2025/sd
25	#23 or #24

Table S4 Characteristics of 22 studies aimed to calibrate or extend the results from pre-existed RCTs

Year	Author	Country	Inclusion and exclusion criteria		Emulation rate (%)	Cancer	Intervention	Comparator	Outcome	Data source
			Number of trials	Number of emulations						
2025	Voelskow et al. [54]	Sweden	16	7	44	Breast	Trastuzumab	No trastuzumab	Multiple	Registry
2025	Jourdain et al. [49]	France	36	3	8	Breast	Trastuzumab deruxtecan	trastuzumab emtansine; tucatinib	Multiple	Health insurance
2025	Fang et al. [42]	China	25	5	20	Leukaemia	Venetoclax + hypomethylating agent	hypomethylating agent	Multiple	EHR
2025	Ganame et al. [48]	France	20	15	75	Stomach	FOLFIRI + bevacizumab	FOLFIRI	OS	MLD
2025	Kulkarni et al. [47]	USA	NA	NA	NA	Breast	PMRT	No PMRT	OS	Registry
2024	Westerberg et al. [2]	Sweden	8	7	88	Prostate	Prostatectomy	Radiotherapy	OS	Registry
2024	Lu et al. [16]	USA	5	5	100	Breast	Breast conserving	Total mastectomy	OS	Registry
2024	Merola et al. [14]	USA	36	3	8	Lung	Pembrolizumab + pemetrexed + platinum	Pemetrexed + platinum	OS	EHR
2024	Chen et al. [26]	China	16	5	31	Lung	Stereotactic ablative radiotherapy	Conventional fractionated radiotherapy	OS	Registry
2024	Lee et al. [17]	Korea	50	3	6	Myeloma	Carfilzomib + lenalidomide + dexamethasone	Lenalidomide + dexamethasone	Safety	Health insurance
2024	Kirkegård et al. [20]	Denmark	28	5	18	Pancreas	Adjuvant chemotherapy	No adjuvant chemotherapy	OS	Registry

Year	Author	Country	Inclusion and exclusion criteria		Emulation rate (%)	Cancer	Intervention	Comparator	Outcome	Data source
			Number of trials	Number of emulations						
2024	Antoine et al. [32]	France	18	13	72	Breast	Palbociclib + fulvestrant	Fulvestrant	OS	MLD
			23	14	61		Palbociclib + letrozole	Letrozole	OS	
			24	13	54		Ribociclib + letrozole	Letrozole	OS	
			21	10	48		Pertuzumab + trastuzumab + docetaxel	Trastuzumab + docetaxel	OS	
			25	8	32		Trastuzumab emtansine	Lapatinib + capecitabine	OS	
			29	11	38		Chemotherapy + bevacizumab	Chemotherapy + placebo	OS	
			11	9	82		Docetaxel + bevacizumab	Docetaxel + placebo	OS	
			22	12	55		Everolimus + exemestane	Exemestane	OS	
2023	Merola et al. [12]	USA	13	13	100	Breast	Palbociclibletrozole	Letrozole	Time-to-next treatment	EHR
2023	Wang et al. [5]	USA	7	5	71	Breast	Mastectomy	Lumpectomy	OS	Registry
2023	Antoine et al. [33]	France	43	11	26	Breast	Paclitaxel + bevacizumab	Paclitaxel	OS	MLD

**Year**	**Author**	**Country**	**Inclusion and exclusion criteria**		**Emulation rate (%)**	**Cancer**	**Intervention**	**Comparator**	**Outcome**	**Data source**
			**Number of trials**	**Number of emulations**						
2022	Ernandez et al. [25]	USA	8	5	63	Bladder	Adjuvant chemotherapy + radiotherapy	Chemotherapy	OS	Registry
2022	Bharadwaj et al. [29]	USA	25	6	24	Bladder	Adjuvant chemotherapy	Observation	OS	Registry
2022	Hou et al. [23]	USA	10	5	50	Colorectum	Laparoscopy	Open colectomy	OS	EHR
2022	Merola et al. [13]	USA	21	11	52	Breast	Palbociclib + fulvestrant	Palbociclib + letrozole	OS	EHR
2021	Boyne et al. [27]	Canada	16	14	88	Colorectum	3 to 5 months of capox or folfox	6 months of capox or folfox	OS	MLD
2020	Bacic et al. [31]	USA	2	2	100	Kidney	Radical nephrectomy with lymph node dissection	Radical nephrectomy without Lymph node dissection	OS	Registry
2020	Petito et al. [8]	USA	5	4	80	Colorectum and pancreas	Fluorouracil	-	OS	MLD
			5	4	80		Erlotinib	-	OS	

RCTs randomized controlled trials, *USA* United States of America, *OS* overall survival, *EHR* electronic health record, *MLD* multiple linked databases, *NA* not available

Table S5 When not to use target trial emulation

Scenario	Example	Why TTE is not ideal	Potential alternative approach
Ill-defined or multi-version treatments	Adaptive chemotherapy regimens, Variable dosing schedules, multi-line therapy sequences	Violates the consistency assumption; Multiple versions of treatment make the causal estimate ambiguous	Policy-based or modified treatment estimate; Incremental propensity-score interventions; Trial emulation restricted to well-defined core components
Extreme or unrealistic strategies	'Treat al' vs. 'treat none'; mandatory early initiation for high-risk patients	Severe positivity/overlap violations; Limited observed data support for counterfactual comparisons	Restrict analyses to regions of empirical equipoise; Use overlap weighting; Pragmatic exposure definitions reflecting real-world constraints
Interference or spillover effects	Hospital-level oncology pathway changes; Multidisciplinary treatment protocols; Antimicrobial stewardship	Violates SUTVA; One patient's outcome may be affected by the treatment of others	Instrumental-variable designs; Cluster-based or natural-experiment approaches
Mediation or effect decomposition	Treatment effect mediated by biomarkers or toxicity	Cross-world assumptions persist; Unmeasured mediator–outcome confounding; TTE alone does not resolve mediation biases	Interventional/organic direct, -indirect or separable effects estimate; Sensitivity analyses for mediator, outcome confounding
Highly time-dependent exposures or delayed treatment initiation	Sequential therapy post-surgery	Ill-defined time zero; Temporal misalignment introduces immortal time bias	Standardized/automated algorithms for defining time zero; Time-varying exposure models; Sensitivity analyses
Effect modification or interaction analyses with multiple moderators	-	Additive vs multiplicative scale issues; Overlap violations when stratifying	Use additive scales (risk difference, RMST), overlap weighting, or hierarchical shrinkage for moderation analysis

TTE target trial emulation, *SUTVA* stable unit treatment value assumption, *RMST* restricted mean survival time

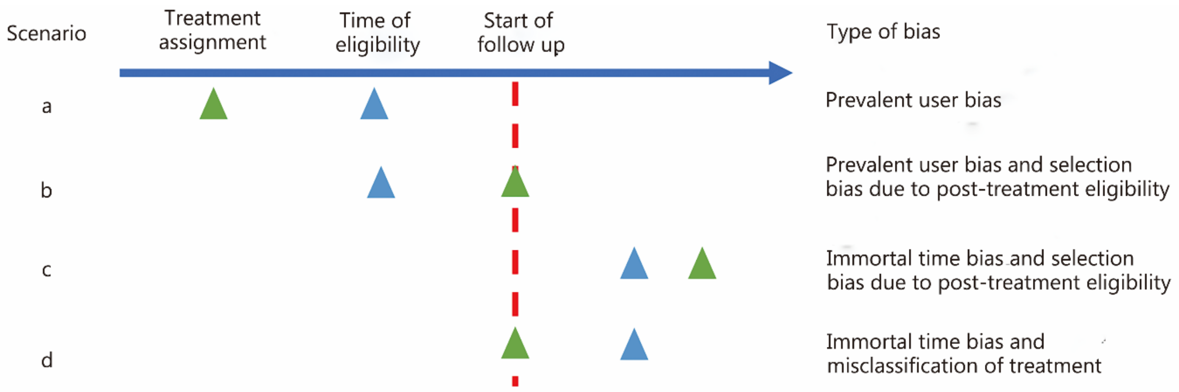

Fig. S1 Biases caused due to asynchronization of eligibility, treatment assignment, and follow-up. The red dashed line indicates the start of follow-up; the green triangle represents the time of treatment assignment; and the blue triangle represents the time of eligibility.

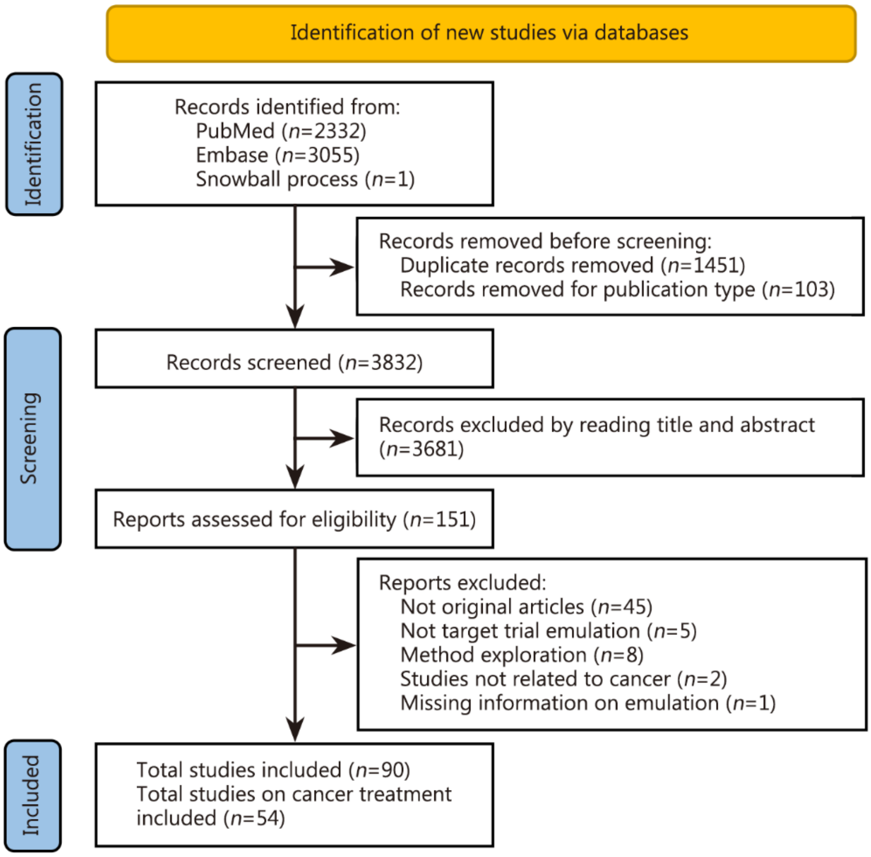

Fig. S2 PRISMA flowchart. PRISMA preferred reporting items for systematic reviews and meta-analyses

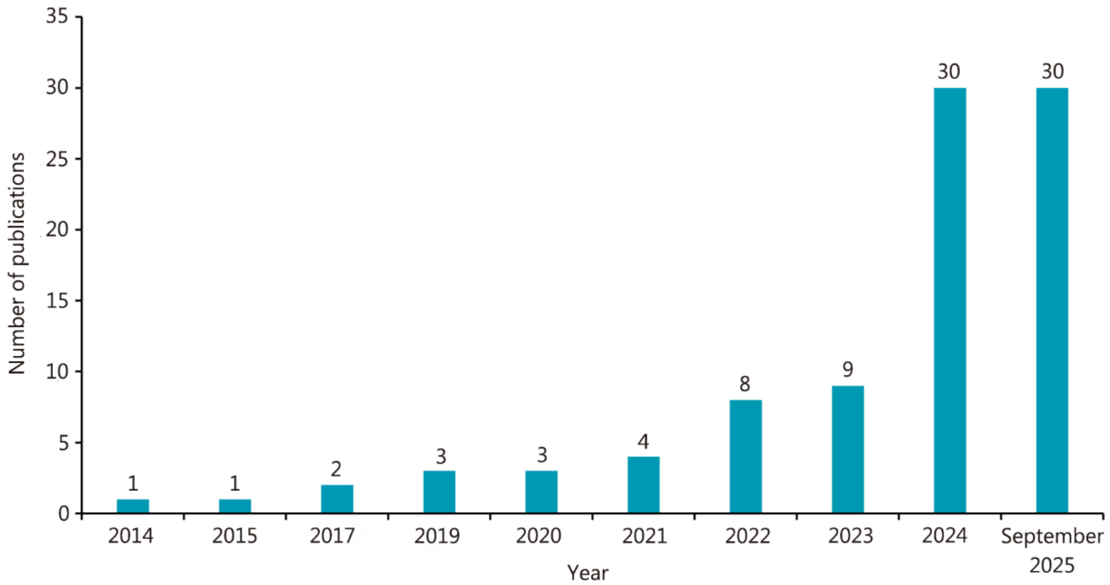

Fig. S3 Time trends in publications using target trial emulation in oncology

Reference

1. Wu CC, Su CC, Lee PT, Chen YH, Hsu CW, Su YC. Comparative effectiveness of primary tumor resection versus chemotherapy in patients with asymptomatic unresectable metastatic colorectal cancer: a retrospective cohort study using the target trial emulation framework. Am J Cancer Res. 2024;14(5):2172-86.
2. Westerberg M, Garmo H, Robinson D, Stattin P, Gedeborg R. Target trial emulation using new comorbidity indices provided risk estimates comparable to a randomized trial. J Clin Epidemiol. 2024;174:111504.
3. Wang J, Zhang S, Yi H, Ma S. Comparative effectiveness analysis of lumpectomy and mastectomy for elderly female breast cancer patients: a deep learning-based big data analysis. Yale J Biol Med. 2023;96(3):327–46.
4. Wang J, Im Y, Wang R, Ma S. Partial hepatectomy and ablation for survival of early-stage hepatocellular carcinoma patients: a bayesian emulation analysis. Life (Basel). 2024; 14(6):661.
5. Smith LH, García-Albéniz X, Chan JM, Zhao S, Cowan JE, Broering JM, et al. Emulation of a target trial with sustained treatment strategies: an application to prostate cancer using both inverse probability weighting and the g-formula. Eur J Epidemiol. 2022;37(12):1205–13.
6. Robak T, Doubek M, Ferrant E, Diels J, Andersone L, Wilbertz S, et al. Overall survival of patients with CLL treated with ibrutinib in the first line compared to second-line ibrutinib after chemotherapy/chemoimmunotherapy. Curr Med Res Opin. 2024;40(8):1369–78.
7. Reitblat C, Fleishman A, Kaplan IA, Stensland KD, D'Amico AV, Olumi AF, et al. Radical prostatectomy versus external beam radiation therapy for high-grade, clinically localized prostate cancer: emulation of a target clinical trial. Urol Oncol. 2021;39(11):785.e1-10.
8. Petito LC, García-Albéniz X, Logan RW, Howlader N, Mariotto AB, Dahabreh IJ, et al. Estimates of overall survival in patients with cancer receiving different treatment regimens: emulating hypothetical target trials in the surveillance, epidemiology, and end results (SEER)-medicare linked database. JAMA Netw Open. 2020;3(3):e200452.
9. Perhanidis JA, Kalilani L, Zimmerman NM, Golembesky A. An emulated target trial case study of real-world overall survival with second-line maintenance niraparib versus active surveillance in patients with recurrent ovarian cancer. Pharmacoepidemiol Drug Saf. 2024;33(9):e70001.
10. Obergfell TTAF, Nydegger KN, Heesen P, Schelling G, Bode-Lesniewska B, Studer G, et al. Improving sarcoma outcomes: target trial emulation to compare the impact of unplanned and planned resections on the outcome. Cancers. 2024;16(13):2443.
11. Morton R, Webb PM, Na R, Obermair A, Farrell R. Mismatch repair status and surgical approach in apparent

early-stage endometrial cancer. Int J Gynecol Cancer. 2024;34(4):535–43.

12. Merola D, Young J, Schrag D, Lin KJ, Alwardt S, Schneeweiss S. Effectiveness research in oncology with electronic health record data: a retrospective cohort study emulating the PALOMA-2 trial. Pharmacoepidemiol Drug Saf. 2023;32(4):426–34.

13. Merola D, Young J, Schrag D, Lin KJ, Robert N, Schneeweiss S. Oncology drug effectiveness from electronic health record data calibrated against RCT evidence: the PARSIFAL trial emulation. Clin Epidemiol. 2022;14:1135–44.

14. Merola D, Campbell U, Lenis D, Schneeweiss S, Wang S, Madsen A, et al. Calibrating observational health record data against a randomized trial. JAMA Netw Open. 2024;7(9): e2436535.

15. Madenci AL, Wanis KN, Cooper Z, Subramanian SV, Haneuse S, Hofman A, et al. Comparison of mortality risk with different surgeon and hospital operative volumes among individuals undergoing pancreatectomy by emulating target trials in US medicare beneficiaries. JAMA Netw Open. 2022;5(3):e221766.

16. Lu Y, Meadows RJ, Gehr AW, Narra K, Bullock J, Ghabach B, et al. Comparative effectiveness of treatment approaches for early invasive breast cancer. Ann Epidemiol. 2024;96:66–72.

17. Lee HK, Jang HY, Kim IW, Oh JM. Target trial emulation of carfilzomib safety among patients with relapsed/refractory multiple myeloma using a nationwide observational data in Korea. J Cancer Res Clin Oncol. 2024;150(5):266.

18. Kwee SA, Wong LL, Sato MM, Acoba JD, Rho YS, Srivastava A, et al. Transarterial radioembolization for hepatocellular carcinoma with major vascular invasion: a nationwide propensity score-matched analysis with target trial emulation. J Vasc Interv Radiol. 2021;32(9):1258-66.e6.

19. Kirkegård J, Ladekarl M, Johannsen IR, Mortensen F. Effect of adjuvant chemotherapy after pancreatectomy in patients with node-negative pancreatic cancer: target trial emulation. Br J Surg. 2024;111(1):znad398.

20. Kirkegård J, Gaber C, Heide-Jørgensen U, Fristrup CW, Lund JL, Cronin-Fenton D, et al. Effect of surgery versus chemotherapy in pancreatic cancer patients: a target trial emulation. J Natl Cancer Inst. 2024;116(7):1072–9.

21. Jochum F, Dumas É, Gougis P, Hamy AS, Querleu D, Lecointre L, et al. Survival outcomes of primary vs interval cytoreductive surgery for international federation of gynecology and obstetrics stage IV ovarian cancer: a nationwide population-based target trial emulation. Am J Obstet Gynecol. 2025;232(2):194.e1-11.

22. Jin ZC, Chen JJ, Zhu XL, Duan XH, Xin YJ, Zhong BY, et al. Immune checkpoint inhibitors and anti-vascular endothelial growth factor antibody/tyrosine kinase inhibitors with or without transarterial chemoembolization

as first-line treatment for advanced hepatocellular carcinoma (CHANCE2201): a target trial emulation study. EClinicalMedicine. 2024;72:102622.

23. Hou J, Zhao R, Cai T, Beaulieu-Jones B, Seyok T, Dahal K, et al. Temporal trends in clinical evidence of 5-year survival within electronic health records among patients with early-stage colon cancer managed with laparoscopy-assisted colectomy vs open colectomy. JAMA Netw Open. 2022;5(6):e2218371.
24. Garcia-Albeniz X, Chan JM, Paciorek A, Logan RW, Kenfield SA, Cooperberg MR, et al. Immediate versus deferred initiation of androgen deprivation therapy in prostate cancer patients with PSA-only relapse. An observational follow-up study. Eur J Cancer. 2015;51(7):817–24.
25. Ernandez J, Kaul S, Fleishman A, Korets R, Chang P, Wagner A, et al. Adjuvant chemotherapy plus radiotherapy versus chemotherapy alone for locally advanced bladder cancer after radical cystectomy. Bladder Cancer. 2022;8(4):405–17.
26. Chen HJ, Cheng WC, Tu CY, Hsia TC, Lin YS, Fang HY, et al. Stereotactic ablative radiotherapy versus conventional fractionated radiotherapy for clinical early-stage non-small-cell lung cancer: a population-based study. Thorac Cancer. 2024;15(24):1779–91.
27. Boyne DJ, Cheung WY, Hilsden RJ, Sajobi TT, Batra A, Friedenreich CM, et al. Association of a shortened duration of adjuvant chemotherapy with overall survival among individuals with stage III colon cancer. JAMA Netw Open. 2021;4(3):e213587.
28. Boyne DJ, Brenner DR, Gupta A, Mackay E, Arora P, Wasiak R et al. Head-to-head comparison of FOLFIRINOX versus gemcitabine plus nab-paclitaxel in advanced pancreatic cancer: a target trial emulation using real-world data. Ann Epidemiol. 2023;78:28–34.
29. Bharadwaj M, Kaul S, Fleishman A, Korets R, Chang P, Wagner A, et al. Adjuvant chemotherapy versus observation following radical cystectomy for locally advanced urothelial carcinoma of the bladder. Urol Oncol. 2022;40(6):274.e15-e23.
30. Basmadjian RB, Lupichuk S, Xu Y, Quan ML, Cheung WY, Brenner DR. Adjuvant ovarian function suppression in premenopausal hormone receptor-positive breast cancer. JAMA Netw Open. 2024;7(3):e242082.
31. Bacic J, Liu T, Thompson RH, Boorjian SA, Leibovich BC, Golijanin D, et al. Emulating target clinical trials of radical nephrectomy with or without lymph node dissection for renal cell carcinoma. Urology. 2020;140:98–106.
32. Antoine A, Pérol D, Robain M, Bachelot T, Choquet R, Jacot W, et al. Assessing the real-world effectiveness of 8 major metastatic breast cancer drugs using target trial emulation. Eur J Cancer. 2024;213:115072.

33. Antoine A, Pérol D, Robain M, Delaloge S, Lasset C, Drouet Y. Target trial emulation to assess real-world efficacy in the Epidemiological Strategy and Medical Economics metastatic breast cancer cohort. J Natl Cancer Inst. 2023;115(8):971–80.

34. Amiot M, Mortier L, Dalle S, Dereure O, Dalac S, Dutriaux C, et al. When to stop immunotherapy for advanced melanoma: the emulated target trials. eClinicalMedicine. 2024;78:102960.

35. Schaufler C, Kaul S, Fleishman A, Korets R, Chang P, Wagner A, et al. Immediate radiotherapy versus observation in patients with node-positive prostate cancer after radical prostatectomy. Prostate Cancer Prostatic Dis. 2024;27(1):81–8.

36. Lemelin A, Maj D, Takemura K, Boyne DJ, Warkentin MT, Brenner DR, et al. CABOSEQ 3-comparison of cabozantinib versus sunitinib following first-line nivolumab- ipilimumab for metastatic renal cell carcinoma: a target trial emulation using real-world data from the international metastatic renal cell carcinoma database consortium (IMDC). Clin Genitourin Cancer. 2025;23(6):102431.

37. Ahn JC, Ng WH, Yeo YH, Kim HS, Wang Y, Trivedi H, et al. Comparative effectiveness of immunotherapy versus lenvatinib in advanced hepatocellular carcinoma: a real-world analysis using target trial emulation. Hepatology. 2025:10.1097/HEP.0000000000001328.

38. Rousseau A, Simon-Tillaux N, Michiels S, Derosa L, Laparra A, Planchard D, et al. Concomitant comedications and survival with first-line pembrolizumab in advanced non-small-cell lung cancer. JAMA Netw Open. 2025;8(9):e2529225.

39. Kuo YH, Lin CY, Wang YC, Lai YL, Kuo YC, Liang JA, et al. Effectiveness of chemotherapy with/without radiotherapy for stage IVb esophageal squamous cell carcinoma: a population-based target trial emulation study. Discov Oncol. 2025;16(1):672.

40. Sorial MN, Han JX, Koh MJ, Boussi L, Li S, Duan R, et al. Forecasting optimal treatments in relapsed/refractory mature T- and NK-cell lymphomas: a global PETAL consortium study. Br J Haematol. 2025;206(6):1664-77.

41. Thill M, Zahn MO, Welt A, Nusch A, Zaiss M, Engelken K, et al. Head-to-head comparison of palbociclib and ribociclib in first-line treatment of HR-positive/HER2-negative metastatic breast cancer with real-world data from the OPAL registry. Int J Cancer. 2025;156(9):1770-82.

42. Fang Q, Fung CY, Wang J, Lai WH, Wong RSM, Kho BCS, et al. Hypomethylating agent versus venetoclax combination: an electronic health records-based target trial emulation among asian elderly patients with newly diagnosed acute myeloid leukaemia in Hong Kong. Ther Adv Hematol. 2025:16:20406207251346914.

43. Zuo H, Vaihenberg E, Singh A, Bal G, Bigras G, Fenton D, et al. Impact of early discontinuation of adjuvant

endocrine therapy on survival in breast cancer: a target trial emulation. Eur J Cancer. 2025:227:115665.
44. Lu TT, Wu CE, Huang TH, Chen JL, Yang CW, Chen HY. Integrating chinese herbal medicine in advanced lung cancer: a multicenter real-world study using target trial emulation. Phytomedicine. 2025:148:157239.
45. Li Y, Zhu L, Ma J, Wang Y, Lin Y, Ye Y, et al. Metronomic chemotherapy of paclitaxel plus cisplatin in patients with metastatic breast cancer: a target trial emulation study. Ther Adv Med Oncol. 2025:17:17588359251384228.
46. Chen PC, Yang AS, Fichera A, Tsai MH, Wu YH, Yeh YM, et al. Neoadjuvant radiotherapy vs up-front surgery for resectable locally advanced rectal cancer. JAMA Netw Open. 2025;8(5):e259049.
47. Kulkarni SE, Patel SA, Jiang C, Schwieger L, Postlewait LM, Arciero CA, et al. Postmastectomy radiation therapy for intermediate-risk breast cancer patients with 0-3 positive axillary lymph nodes: emulating the SUPREMO trial using real-world data. Clin Breast Cancer. 2025;25(5):e655-65.e4.
48. Ganame S, Walter T, Durand A, Lièvre A, Tougeron D, Scoazec JY, et al. Proof of concept and design of an externally controlled trial for patients with gastro-enteropancreatic neuroendocrine carcinomas based on the randomized phase II BEVANEC trial. Eur J Cancer. 2025:225:115450.
49. Jourdain H, Di Meglio A, Mansouri I, Desplas D, Zureik M, Haddy N. Real-world efficacy and safety of trastuzumab deruxtecan versus trastuzumab emtansine and tucatinib as second-line and third-line treatments for HER2-positive metastatic breast cancer: two target trial emulation studies. Lancet Reg Health Eur. 2025:58:101455.
50. Lee J, Kim M, Han HJ, Kim S, Suh HS. Real-world survival outcomes of immune checkpoint inhibitor therapy after standard treatment failure in EGFR-mutated NSCLC: a nationwide cohort study. Lung Cancer. 2025:206:108682.
51. Jochum F, Dumas É, Gougis P, Hamy AS, Querleu D, Lecointre L, et al. Survival outcomes of primary vs interval cytoreductive surgery for international federation of gynecology and obstetrics stage IV ovarian cancer: a nationwide population-based target trial emulation. Am J Obstet Gynecol. 2025;232(2):194.e1-11.
52. Pichler R, Fritz J, Maier S, Hassler MR, Krauter J, D Andrea D, et al. Target trial emulation to evaluate the effect of immune-related adverse events on outcomes in metastatic urothelial cancer. Cancer Immunol Immunother. 2024;74(1):30.
53. Lampa E, Boros M, Berglund A, Wagenius G, Oskarsdottir GN. Timing of adjuvant chemotherapy after surgical resection for non-small cell lung cancer: a target trial emulation using nationwide swedish registry data. Ann Surg Oncol. 2025;32(7):4650-9.
54. Voelskow V, Garcia-Albeniz X, Berglund A, Feychting M, Kurth T, Matthews AA. Trastuzumab in early curative

breast cancer: a target trial emulation benchmarked against two randomized clinical trials. PLoS Med. 2025;22(7):e1004661.

55. Yang Y, Hodge AM, Lynch BM, Dugué PA, Williamson EJ, Jayasekara H, et al. Sustained hypothetical interventions on midlife alcohol consumption in relation to all-cause and cancer mortality: the Australian longitudinal study on women's health. Am J Epidemiol. 2024;193(1):75–86.

56. Yang CT, Yao WY, Yang CY, Peng ZY, Ou HT, Kuo S. Lower risks of cirrhosis and hepatocellular carcinoma with GLP-1RAs in type 2 diabetes: a nationwide cohort study using target trial emulation framework. J Intern Med. 2024;295(3):357–68.

57. Xu W, Chan L, Danaei G, Lu Y, Wan EYF. Long-term statin use and risk of cancers: a target trial emulation study. J Clin Epidemiol. 2024;172:111425.

58. Xu S, Zheng B, Su B, Finkelstein SN, Welsch R, Ng K, et al. Can metformin prevent cancer relative to sulfonylureas? a target trial emulation accounting for competing risks and poor overlap via double/debiased machine learning estimators. Am J Epidemiol. 2025;194(2):512-23.

59. Tuesley KM, Spilsbury K, Webb PM, Pearson SA, Donovan P, Coory MD, et al. Use of an emulated trial to investigate the association between use of nitrogen-based bisphosphonates and risk of epithelial ovarian cancer. Int J Epidemiol. 2024;53(4):dyae108.

60. Tsilidis KK, Capothanassi D, Allen NE, Rizos EC, Lopez DS, van Veldhoven K, et al. Metformin does not affect cancer risk: a cohort study in the U.K. clinical practice research datalink analyzed like an intention-to-treat trial. Diabetes Care. 2014;37(9):2522–32.

61. Truong B, Hornsby L, Fox B, Chou C, Zheng J, Qian J. Effectiveness and safety of direct oral anticoagulants versus warfarin in patients with atrial fibrillation and cancer: a target trial emulation from SEER-medicare database. Cardiovasc Drugs Ther. 2025;39(4):823-35.

62. Sinn DH, Kang D, Park Y, Kim H, Hong YS, Cho J, et al. Statin use and the risk of hepatocellular carcinoma among patients with chronic hepatitis B: an emulated target trial using longitudinal nationwide population cohort data. BMC Gastroenterol. 2023;23(1):366.

63. Park Y, Kang D, Sinn DH, Kim H, Hong YS, Cho J, et al. Effect of lifestyle modification on hepatocellular carcinoma incidence and mortality among patients with chronic hepatitis B. World J Gastroenterol. 2023;29(24):3843–54.

64. Lazzati A, Epaud S, Ortala M, Katsahian S, Lanoy E. Effect of bariatric surgery on cancer risk: results from an emulated target trial using population-based data. Br J Surg. 2022;109(5):433–8.

65. Kraglund F, Christensen DH, Eiset AH, Villadsen GE, West J, Jepsen P. Effects of statins and aspirin on HCC risk in alcohol-related cirrhosis: nationwide emulated trials. Hepatol Commun. 2023;7(1):e0013.

66. Ju C, Lau WCY, Chambers P, Man KKC, Forster MD, Mackenzie IS, et al. Effect of statin treatment on the risk of cancer in patients with heart failure: A target trial emulation study. Pharmacoepidemiol Drug Saf. 2024;33(3):e5775.

67. Guo F, McGee EE, Chiu YH, Giovannucci E, Mucci LA, Dickerman BA. Evaluating recommendation-based dietary and physical activity strategies for prostate cancer prevention: a target trial emulation in the health professionals follow-up study. Am J Epidemiol. 2025;194(2):449-59.

68. García-Albéniz X, Hsu J, Hernán MA. The value of explicitly emulating a target trial when using real world evidence: an application to colorectal cancer screening. Eur J Epidemiol. 2017;32(6):495–500.

69. García-Albéniz X, Hsu J, Bretthauer M, Hernán MA. Effectiveness of screening colonoscopy to prevent colorectal cancer among medicare beneficiaries aged 70 to 79 years: a prospective observational study. Ann Intern Med. 2017;166(1):18–26.

70. Emilsson L, Song M, Ludvigsson JF. Target trial emulation of aspirin after diagnosis of colorectal polyps. Eur J Epidemiol. 2023;38(10):1105–14.

71. Dickerman BA, Giovannucci E, Pernar CH, Mucci LA, Hernán MA. Guideline-based physical activity and survival among us men with nonmetastatic prostate cancer. Am J Epidemiol. 2019;188(3):579–86.

72. Dickerman BA, García-Albéniz X, Logan RW, Denaxas S, Hernán MA. Emulating a target trial in case-control designs: an application to statins and colorectal cancer. Int J Epidemiol. 2020;49(5):1637–46.

73. Dickerman BA, García-Albéniz X, Logan RW, Denaxas S, Hernán MA. Evaluating metformin strategies for cancer prevention: a target trial emulation using electronic health records. Epidemiology. 2023;34(5):690–9.

74. Dickerman BA, García-Albéniz X, Logan RW, Denaxas S, Hernán MA. Avoidable flaws in observational analyses: an application to statins and cancer. Nat Med. 2019;25(10):1601–6.

75. Braitmaier M, Schwarz S, Kollhorst B, Senore C, Didelez V, Haug U. Screening colonoscopy similarly prevented distal and proximal colorectal cancer: a prospective study among 55–69-year-olds. J Clin Epidemiol. 2022;149:118–26.

76. Börnhorst C, Reinders T, Rathmann W, Bongaerts B, Haug U, Didelez V, et al. Avoiding time-related biases: a feasibility study on antidiabetic drugs and pancreatic cancer applying the parametric g-formula to a large German healthcare database. Clin Epidemiol. 2021;13:1027–38.

77. Krebs E, Weymann D, Ho C, Weppler A, Bosdet I, Karsan A, et al. Clinical effectiveness and cost-effectiveness

of multigene panel sequencing in advanced melanoma: a population-level real-world target trial emulation. JCO Precis Oncol. 2025:9:e2400631.

78. McGee EE, Hernán MA, Giovannucci E, Mucci LA, Chiu YH, Eliassen AH, et al. Estimating the effects of lifestyle interventions on mortality among cancer survivors: a methodologic framework. Epidemiology. 2025;36(5):705-18.

79. Guo F, McGee EE, Chiu YH, Giovannucci E, Mucci LA, Dickerman BA. Evaluating recommendation-based dietary and physical activity strategies for prostate cancer prevention: a target trial emulation in the health professionals follow-up study. Am J Epidemiol. 2025;194(2):449-459.

80. Abiodun AT, Ju C, Welch CA, Lai J, Tyrer F, Chambers P, et al. Fluoropyrimidine chemotherapy and the risk of death and cardiovascular events in patients with gastrointestinal cancer. JACC CardioOncol. 2025;7(4):345-56.

81. Wang L, Wang Q, Li L, Kaelber DC, Xu R. Glucagon-like peptide-1 receptor agonists and pancreatic cancer risk: target trial emulation using real-world data. J Natl Cancer Inst. 2025;117(3):476-85.

82. Chen N, McGee EE, Nethery RC, Mucci LA, Dickerman BA. Guideline-based physical activity and health-related quality of life among prostate cancer survivors: a target trial emulation in the health professionals follow-up study. Am J Epidemiol. 2025:kwaf117.

83. Gamborg M, Grand MK, Grell K, Rosthøj S, Pedersen-Bjergaard U, Torp-Pedersen C, et al. Long-term cancer risk in users of GLP-1 agonists in Denmark: a nationwide emulated trial. Lancet Reg Health Eur. 2025:55:101346.

84. To SY, Lee CH, Chen YH, Hsu CL, Yang HW, Jiang YS, et al. Psoriasis risk with immune checkpoint inhibitors. JAMA Dermatol. 2025;161(1):31-8.

85. Lim Y, Kim E, Hwang E, Kim K, Son Y, Lee SY, et al. Real-world evidence on the additional cancer risk reduction of ezetimibe: a 10-year nationwide retrospective cohort study emulating a target trial. J Cancer Res Clin Oncol. 2025;151(10):271.

86. Wu CC, Su CC, Chang YC, Lee PT, Su YC. Reduced risks of colorectal cancer with GLP-1RAs in type 2 diabetes: a nationwide cohort study using a target trial emulation framework. Diabetes Metab. 2025;51(6):101695.

87. Kuranishi F, Miyazaki T, Tagashira T, Fujii A, Yuba M, Miyake I, et al. Thirty-five-year follow-up real-world data revealed the efficacy of autologous formalin-fixed tumor vaccine on metastatic breast cancer-a target trial emulation. Clin Breast Cancer. 2025;25(8):e1011-22.e5.

88. Kang D, Kim N, Kim H, Lee AY, Park J, Kim S, et al. Emulating trial to evaluate the effectiveness of routine supportive care on mortality among cancer patients experiencing distress at the time of diagnosis. J Affect Disord.

2024;354:519–25.

89. Ehrencrona C, Li Y, Angenete E, Haglind E, Franzén S, Grimby-Ekman A, et al. Do beta-blockers reduce negative intrusive thoughts and anxiety in cancer survivors? - An emulated trial. BMC Cancer. 2024;24(1):447.

90. Buranupakorn T, Thangsuk P, Patumanond J, Phinyo P. Emulation of a target trial to evaluate the causal effect of palliative care consultation on the survival time of patients with hepatocellular carcinoma. Cancers. 2021;13(5):1–15.
